# The Relationship Between Obesity and Cancer: Epidemiology, Pathophysiology, and the Effect of Obesity Treatment on Cancer

**DOI:** 10.3390/curroncol32060362

**Published:** 2025-06-19

**Authors:** Yasmin Ingram, Oluwasegun Olujide, Nabiha Sheikh, Alice Robinson, Jan Hoong Ho, Akheel A. Syed, Safwaan Adam

**Affiliations:** 1 The Christie NHS Foundation Trust, Manchester M20 4BX, UK; 2 Western General Hospital, Edinburgh EH4 2XU, UK; 3 Salford Royal NHS Foundation Trust, Salford M6 8HD, UK; 4 Faculty of Biology, Medicine and Health, University of Manchester, Manchester M13 9PL, UK

**Keywords:** obesity, cancer, obesity-related cancer

## Abstract

There is growing evidence relating to the risk of cancer in people with obesity. Obesity is already established as one of the strongest predisposing factors to cancer, and ‘obesity-related’ cancers have been defined in previous studies. In this review article, we examine the epidemiological relationship and describe the potential pathophysiological mechanisms that underpin the association between obesity and cancer. These include hormonal and growth factors that are in abundance in persons living with obesity and thereby increase cancer risk. Additionally, the increased disposition towards chronic inflammation in obesity also confers cancer risk. We also examine the impact of obesity on cancer treatment outcomes, focusing on surgery, chemotherapy, and immunotherapy. Conversely, we underline the impact of weight loss on cancer risk by examining different weight loss strategies.

## 1. Background

Obesity is a complex chronic disease defined by excessive fat deposits that can impair health. It is typically evaluated using four morphometric measures: the Body Mass Index (BMI), calculated as weight in kilograms divided by height in metres squared, and, less frequently, waist circumference, waist-to-hip ratio, and waist-to-height ratio. The World Health Organisation formally defines obesity as a BMI of 30 kg/m^2^ or greater [1]. Recently, however, there have been calls to redefine obesity based on the clinical impact of increased adiposity [2], though there is currently no evidence supporting improved risk prediction with this approach. In contrast, the concept of metabolically healthy obesity, characterised by the absence of insulin resistance, has been widely tested for this purpose [3] and may influence future classifications.

Obesity has become a significant global health threat, with almost 39% of adults classified as overweight and over 800 million of them considered clinically obese as of 2020. If this current trajectory is maintained, almost half of the world’s population could be overweight or obese by 2030 [1]. This carries profound implications for various diseases, in particular cancer, on which this article will draw its primary focus.

**Figure 1:**
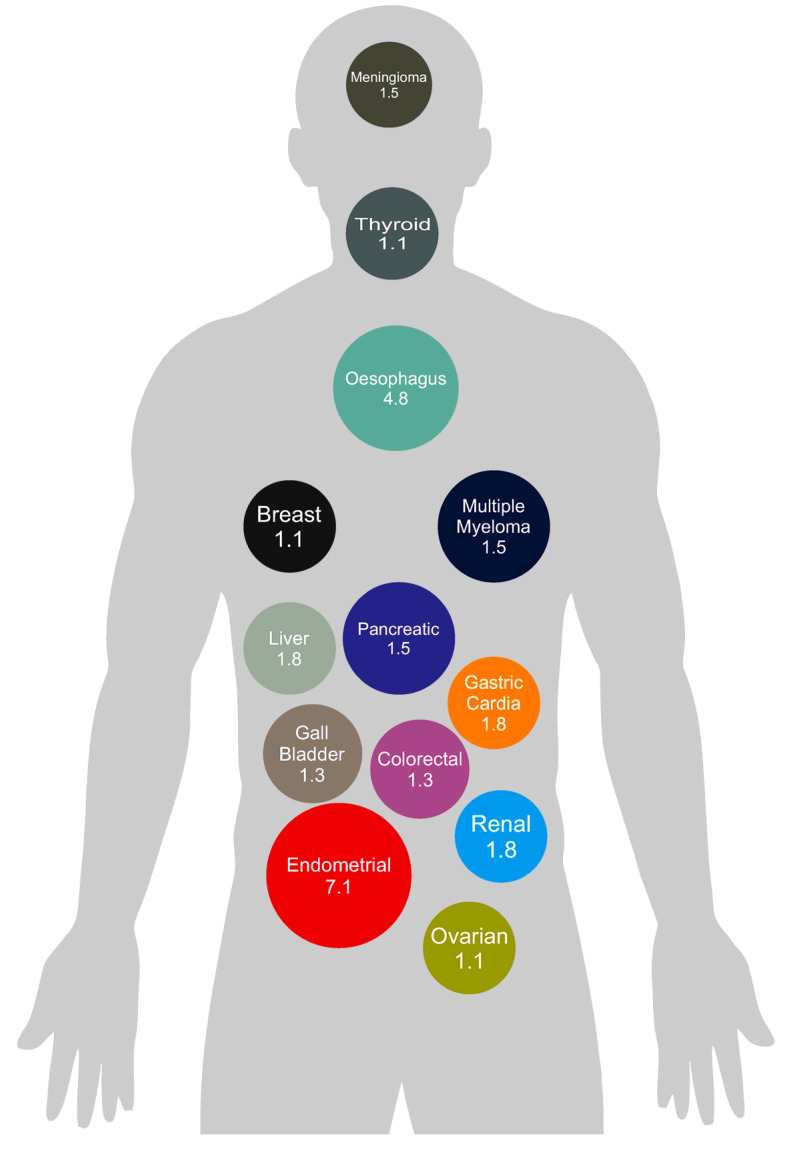
Summary of observational epidemiologic evidence on the association between obesity and cancer risk by cancer site. Figure 1 demonstrates the strength of the association between obesity and the development of 13 different cancers. The values represented are based on relative risk ratio and derived from data presented in the publication by Lauby-Secretan et al. [3].

The link between obesity and cancer cannot be understated. A review of the available literature reveals a substantial amount of epidemiological research which demonstrates a significant association between obesity and increased cancer risk for multiple cancer sites (Figure 1). This evidence is not only abundant but also robust, comprising multiple meta-analyses which draw from more than 1000 studies globally [4].

Excess weight and obesity are now the UK’s largest modifiable cause of cancer after smoking. It is estimated that approximately 4–8% of all cancers are associated with obesity [5], with some individual cancer types having up to a 32% association, further underscoring the urgent need to address this issue [5]. The 2020 World Cancer Report by the International Agency for Research on Cancer (IARC) reported at least ‘sufficient’ evidence linking obesity to a heightened risk of 13 types of cancer, including those of the breast (postmenopausal), colon, endometrium, ovary, kidney, liver, gall bladder, gastric cardia, oesophagus, and pancreas, alongside thyroid cancer, multiple myeloma, and meningioma [4].

The relationship between obesity and cancer risk is complex and has been shown to vary amongst different patient demographic groups. This association has been demonstrated to be more pronounced in women than in men [6], alongside being more marked in older patients compared to their younger counterparts [7]. Furthermore, the available evidence suggests that obesity’s impact on cancer risk may differ according to race and ethnicity. For instance, research suggests Asian women have a greater risk of breast cancer related to obesity compared to women of other ethnic backgrounds [7]. This is believed to be partly attributable to variations in body fat distribution among different ethnic groups, as a larger waist circumference is now accepted as a risk factor for many cancers, irrespective of body size [8]. Furthermore, the duration of obesity is also an important risk factor for the development of cancer, with longer duration conferring greater risk [9].

In addition, it is important to recognise that BMI, as a measure of fat mass, may underestimate the fat mass of taller individuals, who typically have a higher fat mass for the same BMI. This discrepancy not only increases the risk of type 2 diabetes [10] but may also contribute to elevated cancer risk. In fact, increased height has also been independently associated with a higher risk of certain cancer types [11].

Adipose tissue itself is composed predominantly of white adipose tissue (WAT), alongside smaller amounts of brown adipose tissue (BAT) and beige adipocytes which have characteristics of both WAT and BAT [12]. While WAT’s primary function is that of energy storage, and that of BAT is thermogenesis [12], both secrete bioactive molecules including adipokines and cytokines, as well as hormones such as leptin, adiponectin, and resistin. These have all been demonstrated to influence the physiological processes that underpin cancer risk in obesity [13,14,15]. In addition to adipocytes, adipose tissue comprises various other cell types, including immune cells, fibroblasts, endothelial cells, and preadipocytes, which also contribute to its roles in endocrine signalling and inflammation [16]. Adipose tissue may also be classified by anatomical distribution, with visceral fat being more metabolically active and thereby more strongly associated with cancer risk [17]. Of note, ageing is associated with a shift in fat distribution from subcutaneous to visceral depots, a change which contributes to increased cancer susceptibility later in life [18].

Economic growth in low- and middle-income countries (LMICs) is highly predictive of increases in BMI [19]. The current economic boom in LMICs brings to the fore the growing issues of the double burden of malnutrition, where rising obesity rates coexist alongside persistent micronutrient deficiencies. This dual burden increases the risk of non-communicable diseases, including cancer [20]. This presents a potential opportunity for prospective studies to better understand the effects of obesity on cancer and subsequently inform future interventions. With the prevalence of obesity rising, cancer incidence worldwide is set to substantially increase. This brings to the fore an urgent need for public health initiatives directed towards obesity prevention and management.

## 2. Pathophysiology

**Figure 2:**
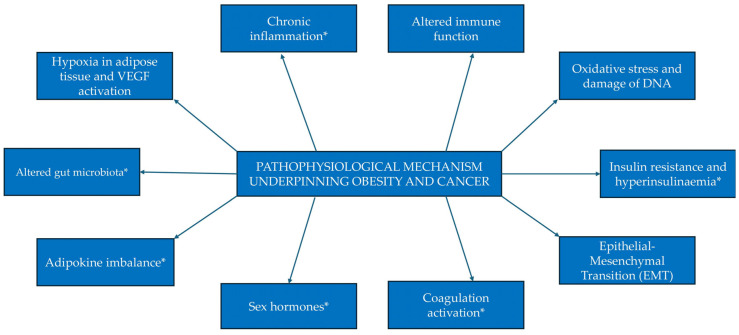
Proposed mechanisms of the pathophysiology underpinning the relationship between obesity and cancer. Mechanisms denoted with an asterisk (*) have been expanded upon below.

Whilst the exact mechanisms underlying the observed increased risk of cancer with underlying obesity have yet to be elucidated, various hypotheses have been explored in the literature (Figure 2).

### 2.1. Sex Hormones

It has been hypothesised that increased levels of bioavailable testosterone and oestrogen in patients with raised BMI may explain the connection between obesity and elevated cancer risk.

Adipocytes are typically rich in the aromatase enzyme, responsible for converting androgens into oestrone and oestradiol. Consequently, in the given context of obesity, as body fat rises, levels of oestrogen increase [21]. This is significant because data suggests that highly active unbound forms of these hormones are linked to a higher risk of hormone-sensitive cancers [10].

Hormones influence cancer risk primarily by regulating cell proliferation and cell survival rates [22]. Oestrogen, through oestrogen receptors (ERs), and testosterone (along with its more potent derivative, dihydrotestosterone), via androgen receptors (ARs), exert their tumorigenic effects through the activation of key signalling pathways [23,24]. Oestrogen and testosterone, when bound to their respective receptors located in the cytoplasm or nucleus of target cells, enhance the expression of cyclins (particularly cyclin D1) which in turn activate cyclin-dependent kinases, leading to uncontrolled cell division [25,26]. These hormones can also activate another important signalling cascade—the mitogen-activated protein kinase (MAPK)/extracellular signal-regulated kinase (ERK) pathway—which promotes cell proliferation, migration, and invasion [23]. Additionally, the Phosphoinositide 3-Kinase (PI3K)/protein kinase B (AKT)/mechanistic target of rapamycin (mTOR) pathway plays a crucial role in cancer cell growth and survival [23,27]. The activation of this pathway by oestrogen and testosterone can also result in unchecked cell proliferation and the inhibition of apoptosis. Other pathways activated by these hormones include the nuclear factor kappa B (NF-kB), Wnt/β-catenin, and Janus kinase (JAK)/signal transducer and activator of transcription (STAT) pathways [27,28].

Observational studies and RCTs suggest that women considered at risk of breast cancer who engage in higher levels of physical activity demonstrate a significant reduction in these unbound sex hormones, alongside possessing higher levels of sex hormone binding globulin (SHBG), which reduces their bioavailability [29]. This is regardless of menopausal status, although existing evidence is more robust in post-menopausal women [29]. These beneficial changes observed in the endogenous hormone profile are primarily thought to be mediated by body fat loss [29].

Epidemiological studies examining the relationship between androgen levels and tumorigenesis have yielded mixed results. In men, increased adiposity has been demonstrated to correlate with lower levels of testosterone [30]. Conversely, in women, obesity has been shown to raise androgen levels [31]. Some research indicates that androgens may play a role in tumorigenesis and the regulation of cell growth in prostate cancer [32], whilst other studies suggest that the androgen-signalling pathway influences breast cancer carcinogenesis [33]. However, in both areas of research, existing evidence is conflicting and inconclusive [33,34].

### 2.2. Insulin and IGF

Excess body fat, particularly central adiposity, is strongly associated with elevated insulin resistance. When blood glucose levels are persistently raised, the pancreas secretes excess insulin, resulting in hyperinsulinaemia. This lowers the level of Insulin-like Growth Factor-Binding Proteins (IGF-BPs), which subsequently raise the levels of bioactive IGF-1 [35]. Increased levels of both insulin and IGF-1 can stimulate tumorigenic intracellular processes mediated by insulin and IGF-1 receptors, respectively [35]. Overstimulation by these molecules is associated with an increased risk of various malignancies, including those of the breast, prostate, and colorectum [36]. Furthermore, persistent activation of these pathways has also been associated with more aggressive cancer behaviour and reduced treatment sensitivity [37,38].

In post-menopausal women, this pathway also influences the bioavailability of sex hormones. Persistent hyperinsulinaemia decreases SHBG whilst increasing circulating oestrogens and androgens, which may further contribute to tumorigenesis [39].

Physical activity, both independently and through its effects on adipose tissue, has been shown to lower plasma insulin and enhance insulin sensitivity and glucose metabolism. Conversely, data regarding the effect of physical activity on IGF-1 and IGFBP-3 remains mixed. Whilst observational studies suggest this lowers levels of these molecules, randomised trials have not consistently reproduced such results, suggesting that reductions in IGF-1 bioavailability may not mediate the process thought to underpin the relationship between obesity and cancer risk [40]. Interventions aimed at reducing sedentary behaviour in adults, however, have demonstrated small but statistically significant reductions in insulin levels [41].

### 2.3. Chronic Inflammation

White adipose tissue (WAT), particularly when expanded in the context of obesity, functions as an active endocrine organ secreting a range of pro-inflammatory cytokines, chemokines, and adipokines speculated to contribute to elevated cancer risk in obesity [13].

As adipose tissue expands and exceeds its blood supply, it gives rise to hypoxic conditions, which induce adipocyte stress and cell death. This results in a heightened production of monocyte chemoattractant protein-1 (MCP-1) [42]. MCP-1 stimulates the proliferation of macrophages which encircle the dying adipose cells they engulf, forming a histological arrangement referred to as crown-like structures (CLSs) [37]. The inflammatory environment created by these structures may enhance tumorigenic processes, including increased cell proliferation and resistance to apoptosis, thus exacerbating cancer progression [43]. This is also thought to contribute to the increased aggression observed in obesity-related cancers, including earlier metastatic spread and worse prognosis, as demonstrated in cohorts of breast and prostate cancer patients [37,38].

**Figure 3:**
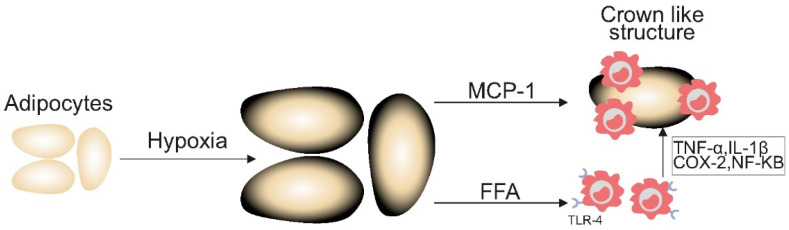
Inflammatory cytokines in obesity increasing the risk of cancer. Figure 3 demonstrates the inflammatory mechanisms underpinning the heightened risk of cancer in people with excess adiposity. MCP-1: monocyte chemoattractant protein-1; TNF-α: tumour necrosis factor-alpha; IL-1β: interleukin-1 beta; COX-2: cyclooxygenase-2; NF-KB: nuclear factor kappa B; TLR-4: toll-like receptor 4; FFA: free fatty acid.

Simultaneously, free fatty acids (FFAs) released from damaged adipocytes can activate toll-like receptor 4 (TLR4) on macrophages, leading to an increased expression of pro-inflammatory genes such as tumour necrosis factor-alpha (TNF-α), interleukin-1 beta (IL-1β), and cyclooxygenase-2 (COX-2) via increased nuclear factor kappa B signalling. This process further perpetuates this cycle of inflammation with elevated levels of pro-inflammatory mediators consistently linked to the presence of CLS in visceral fat [44] (Figure 3).

Early studies have shown that the presence of CLS on tissue biopsy has been associated with reduced overall survival in both breast and tongue cancer sub-types. While initial evidence suggests WAT inflammation has a significant negative impact on disease trajectory in these cases, more studies with robust sample sizes are needed to further elucidate this relationship [37].

These inflammatory changes in adipose tissue may also be reflected in measurable clinical markers. Phase angle (PhA), derived from bioelectrical impedance analysis, has demonstrated potential as a non-invasive indicator of cellular health and integrity in obesity [45]. By assessing both lean and fat mass, PhA allows for a more nuanced evaluation of obesity than BMI alone. Notably, low PhA values have been associated with increased inflammation, sarcopenia, and worse outcomes in obesity-related cancers, affirming its potential as a prognostic biomarker [45,46].

### 2.4. Adipokine Imbalance

In addition to inflammatory mediators, adipose tissue also secretes other adipokines, such as leptin, resistin, and adiponectin, which crucially regulate metabolic processes, inflammation, and cell signalling [14,15]. Depending on the specific type, adipokines can either promote or inhibit tumorigenesis and metastases. However, their imbalance in obesity favours tumorigenesis [15].

Leptin levels are typically elevated in individuals with obesity, leading to an upregulation of pro-inflammatory cytokines like TNF-a and IL-6 [14,15]. Leptin activates signalling pathways such as JAK/STAT3, PI3K/AKt, and MAPK, resulting in an uncontrolled cell proliferation, inhibition of apoptosis, and metastasis promotion [14,15]. Furthermore, leptin can enhance invasion and distant metastases via the activation of the TLR4/Src/EGFR/PI3K/NF-κB signalling pathway [14,15]. Leptin can also induce the expression of vascular endothelial growth factor (VEGF), thus promoting angiogenesis, which is essential for cancer growth [14]. Additionally, leptin has also been implicated in the epithelial-to-mesenchymal transition (EMT) and gain of cancer stemness via the activation of the TGF-b, NOTCH, Hedgehog, and Wnt pathways [14,15]. Similarly, resistin, a member of the resistin-like molecule family (RELMs), is elevated in obesity [14,15]. Resistin binds to receptors such as the transmembrane Toll-like receptor 4 (TLR4) and adenylyl cyclase-associated protein 1 (CAP1) to activate several signalling pathways which promotes tumorigenesis [14,15]. Resistin has been shown to inhibit apoptosis through the activation of the MAPK/ERK, JAK/STAT, and AKt/PI3K pathways [14,15]. In contrast, adiponectin levels are reduced in obesity and counteract the effects of leptin [15]. Adiponectin activates the AMPK pathway to suppress angiogenesis, inhibit cell proliferation, and promote apoptosis in cancer cells [15].

### 2.5. Coagulation System

Pro-inflammatory cytokines (such as TNF-a and IL-6) derived from chronic low-grade inflammation in obesity can activate the coagulation system, leading to the release of clotting factors, which are intricately linked with tumour growth and progression [47,48].

Thrombin has been implicated in the proliferation and enhanced survival of cancer cells through activation of protease-activated receptors (PARs) on these cells [47]. Additionally, thrombin plays a role in tumour cell invasion by activating matrix metalloproteinases (MMPs), which degrade the extracellular matrix [47]. Thrombin also promotes angiogenesis through upregulating VEGF expression [47]. These coagulation-related changes may contribute to a more aggressive disease course, with increased potential for invasion, immune evasion, and dissemination to distant sites [49].

Fibrinogen, a soluble plasma protein, is converted into insoluble fibrin by thrombin [48]. Fibrin mesh helps create a physical barrier that shields tumour cells from immune cells like cytotoxic T lymphocytes (CTLs) and natural killer (NK) cells, thus aiding tumour cell survival [48,50]. Fibrinogen also enhances tumour metastases by binding to integrins on cancer cell surface, promoting adhesion and migration [50]. Cancer cells can also interact with fibrin mesh, which helps them detach from primary tumour sites and spread to distant sites [50]. Furthermore, fibrinogen promotes angiogenesis through stimulating the release of VEGF [50]. Studies have shown that high levels of fibrinogen correlate with tumour progression and the likelihood of metastases and are associated with poor prognosis [48,51].

Tissue factor (TF), which is overexpressed in obesity and certain cancers (such as breast, ovarian, and colon cancers), promotes tumour cell growth and survival by activating pathways like MAPK and PI3K/AKT [47,50]. It also supports angiogenesis through the activation of VEGF and plays a role in tumour invasion and metastasis [47,50]. The critical role of TF in tumorigenesis has been highlighted using TF-targeted therapies as potential cancer treatments.

### 2.6. Gut Microbiota

The gut microbiota are an ecosystem of micro-organisms living in the human gastrointestinal tract, essential for immune system function, metabolism, and inflammation [52]. In individuals with obesity, an imbalance in the composition of the gut microbiota, known as dysbiosis, is commonly observed [52]. Dysbiosis has been linked to various health conditions, with a growing body of evidence indicating that it plays key roles in tumorigenesis through multiple mechanisms, including chronic low-grade inflammation, altered metabolism, impaired immunity, and the production of carcinogenic metabolites [52].

Obesity-related gut dysbiosis is typified by an overgrowth of pro-inflammatory bacteria, such as proteobacteria and firmicutes [52]. These bacteria trigger the release of inflammatory cytokines, which promote chronic low-grade inflammation [52].

Gut microbiota also influence the metabolism of bile acids, which are necessary for fat digestion [52,53]. An altered microbiota composition can thus alter bile acid metabolism, resulting in the generation of more secondary bile acids such as deoxycholic acid (DCA) [53]. DCA can directly damage DNA of intestinal epithelial cells or indirectly by activating inflammatory pathways to cause cancer, notably colorectal cancer [53].

Anti-inflammatory bacteria help metabolise dietary fibre into short-chain fatty acids (SCFAs), which have anti-inflammatory and anti-cancer properties [52]. The paucity of these beneficial bacteria in obesity lead to a reduction in gut SCFAs, resulting in weakened immune surveillance and the promotion of tumorigenesis [52].

Additionally, dysbiosis can contribute to tumour development through the production of toxins, such as hydrogen sulphide, which directly damage DNA [52,53]. Furthermore, the altered microbiota in obesity can impede the activity of immune cells such as NK cells leading to a suppression of the body’s ability to detect and eliminate cancer cells [52,53]. Unhealthy microbiota can also disrupt the gut epithelium, leading to ‘leaky gut’, which can allow toxins and other harmful substances into systemic circulation, triggering systemic inflammation and contributing to cancer initiation [52]. This inflammation, coupled with impaired immune surveillance, may contribute to the more aggressive disease trajectories seen in obese individuals with colorectal and other gastrointestinal malignancies [54].

Whilst these mechanisms have been associated with obesity, it raises the question of whether any intervention designed to reduce these risks should aim to address all of these simultaneously.

## 3. Obesity and Cancer Outcomes

Obesity not only increases the risk of developing cancer but also influences cancer outcomes.

### 3.1. Obesity and Cancer Survival and Recurrence

Obesity is associated with worse survival outcomes and a higher likelihood of cancer recurrence [5,55]. A meta-analysis of 6.3 million participants across 203 studies found an increased risk of both overall and cancer-specific mortality in breast, colorectal, prostate, and pancreatic cancer [55]. Relatedly, there was a 13% increase in risk of cancer recurrence in patients with obesity [55].

Some studies, however, suggest that being overweight or obese may be linked to better treatment responses and improved survival outcomes in cancer patients, a phenomenon referred to as the ‘obesity paradox’ [56,57]. Certain studies, including a meta-analysis, have reported lower lung cancer and melanoma mortality rates in patients with obesity [55]. Moreover, pooled evidence suggests that patients with obesity undergoing lung cancer surgery may experience better in-hospital outcomes and longer-term survival [58]. Putative mechanisms for these findings include increased energy reserve, pharmacological factors, and possible detection bias. However, the strength of this evidence is limited, as it is based on 25 observational studies, 23 of which are retrospective, and there is a paucity of high-quality randomised controlled trials [58]. The obesity paradox, originally described in patients with heart failure, has also been reported in various other disease states. However, the supposed paradox is considered to be a statistical artefact resulting from collider stratification, a common source of selection bias in epidemiologic research [59,60,61]. In heart failure, for instance, a recent study has demonstrated that there is no evidence of an obesity paradox when using waist-to-height ratio rather than BMI, and, conversely, individuals with higher levels of body fat were more likely to be hospitalised due to heart failure [62].

### 3.2. Obesity and Cancer Treatment-Related Adverse Effects

Obesity also exacerbates cancer treatment-related side effects (Table 1). Lymphoedema, a well-documented complication of breast cancer surgery, has been shown to increase in proportion to the patient’s BMI [63]. Several factors contribute to this, including increased tissue mass and fluid volume, impaired lymphatic drainage due to excess adipose tissue, compression of lymphatic vessels by excess fat, direct damage and scarring of lymphatics from chronic inflammation, and increased incidence of surgical site infections [64]. Chemotherapy-related peripheral neuropathy is another notable side effect, which studies have found to be more common in patients with obesity [65,66]. This may be explained by several factors, including the increased oxidative stress in individuals with obesity and the release of free radicals from chemotherapy agents, both of which can damage nerves [5]. Additionally, comorbid conditions such as diabetes mellitus and peripheral vascular disease, which are more prevalent in patients with obesity, may predispose them to or exacerbate peripheral neuropathy [67].

Certain chemotherapy agents are well-known causes of cardiomyopathy. A meta-analysis showed that obesity was linked to a 47% increased risk of cardiotoxicity in women with early breast cancer who received trastuzumab and anthracyclines [68]. Obesity has also been found to exacerbate radiation-related toxicities. A systematic review and meta-analysis revealed an 11% increase in the risk of radiation-related dermatitis in breast cancer patients with a BMI greater than 25 kg/m^2^ [69] Additionally, obesity can negatively impact long-term treatment outcomes and quality of life after radiation, particularly by increasing the risk of urinary and bowel incontinence in patients with prostate and gynaecological cancers [70,71]. Some of the mechanisms underlying these toxicities include excess skin folds leading to moisture accumulation, excessive friction, and a higher risk of infection. Additionally, altered vascularity and hypoxia in fatty tissues can delay tissue repair. Furthermore, obesity itself adds to the challenge of radiotherapy planning and delivery [71,72]. Obesity is also associated with a higher incidence of cancer-related surgical complications [67]. While some studies report no significant link between BMI and major surgical complications or short-term mortality [73,74], obesity has been associated with a greater risk of minor complications, such as infections and wound dehiscence [73].

**Table 1:** Table summarising the impact of obesity in relation to cancer treatment-related side effects.

Cancer Treatment Side Effect	Impact of Obesity	Underlying Mechanism
Lymphoedema	Increased risk proportional to BMI [63]	- Increased tissue mass and fluid volume [64] - Impaired lymphatic drainage [64] - Scarring of lymphatics from chronic inflammation [64] - Increased incidence of surgical site infections [64]
Chemotherapy associated peripheral neuropathy	More common in patients with obesity [65,66]	- Increased oxidative stress [5] - Free radical release from chemotherapy agents [5] - Comorbid conditions (diabetes mellitus and peripheral vascular disease) predispose to or exacerbate neuropathy [67]
Cardiotoxicity	47% increased risk in women receiving Trastuzamab and anthracyclines [68]	- Adiponectin downregulation exacerbating left ventricular dysfunction after doxorubicin administration [68] - Mechanism with Trastuzamab unclear [68]
Radiation-related dermatitis	11% increased risk in those with BMI > 25 kg/m^2^ [69]	- Excess skin folds, friction, and moisture accumulation [71,72] - Higher risk of infection [71,72] - Altered vascularity and hypoxia in fatty tissues delay tissue repair [71,72] - Difficult radiotherapy planning and delivery [71,72]
Urinary and Bowel Incontinence (post-radiation)	Increased risk in prostate and gynaecological cancers [70,71]	Excess skin folds, friction, and moisture accumulation [71,72] - Higher risk of infection [71,72] - Altered vascularity and hypoxia in fatty tissues delay tissue repair [71,72] - Difficult radiotherapy planning and delivery [71,72]
Surgical complications	Increased risk of infection and wound dehiscence [73]	- Delayed tissue repair [71,72] - Excess adipose tissue [71,72]

## 4. Efficacy and Toxicity of Cancer Treatments in Patients with Obesity

### 4.1. Chemotherapy

Traditional chemotherapy remains a cornerstone in the treatment of many cancers. Chemotherapy targets rapidly dividing cells, including cancerous and healthy cells, resulting in distinct toxicity profiles [72]. Clinical outcomes after chemotherapy in patients with obesity compared to their normal-weight counterparts are mixed. For instance, some studies in non-small-cell lung cancer patients have suggested that individuals with obesity receiving chemotherapy may experience better overall survival than those with normal weight [75,76]. However, other research found no significant difference in outcomes regardless of BMI [77], while another study linked obesity to poorer survival [78]. The poorer outcomes observed in some studies may be attributed to suboptimal chemotherapy dosing due to dose adjustment or capping in patients with obesity to minimise the risk of excessive toxicity [79]. Research has shown that patients with obesity who receive dose reductions below what would be appropriate based on their actual body weight tend to have worse disease-free and overall survival rates [80,81]. Reliable data also indicate that patients with obesity who receive full chemotherapy doses do not experience higher toxicity levels compared to those with a healthy weight [79]. As a result, the American Society of Clinical Oncology (ASCO) recommends using full, weight-based chemotherapy doses for patients with obesity [82]. Importantly, sarcopenic obesity has consistently been linked to a higher risk of toxicity and poorer treatment outcomes [83,84].

### 4.2. Molecular Targeted Therapies

Molecular targeted therapies are used in specific cancers where the modulation of a specific molecular pathway can provide a precise method of treating neoplasia [85]. Although there is a general paucity of evidence surrounding the impact of obesity on the efficacy or toxicity of these treatments, some studies have shown an important effect of adiposity. A very recent subanalysis of the APHINITY trial, in which patients were treated with a HER2 receptor antagonist in combination with chemotherapy, showed that patients with a BMI > 25 kg/m^2^ demonstrated a shorter invasive disease-free and overall survival compared to patients with a BMI < 25 kg/m^2^ (adjusted hazard ratios 1.27 and 1.38, respectively). This was maintained even after adjusting for chemotherapy discontinuation, which was more prevalent in the sub-cohort with a higher BMI [86]. Contrastingly, in colorectal cancer, a previous systematic review and meta-analysis (nine studies) by Lang et al. showed that there was no significant difference in overall or progression-free survival between obese and non-obese individuals at 1, 2, and 5 years after treatment with bevacizumab combined with chemotherapy. Interestingly, in this analysis, at the 6-month timepoint after treatment, there was a slight short-term survival benefit seen in patients with obesity compared to those without (risk ratio 0.97; *p* < 0.05) [87]. A previous retrospective study in patients with lung cancer (*n* = 40) treated with ALK inhibitors, stratified by BMI (>25 vs. <25 kg/m^2^), showed that patients with a higher BMI had better overall and progression-free survival [88]. However, due to the study being retrospective and of a small sample size, further data are needed to examine this potential relationship.

### 4.3. Immune Checkpoint Inhibitors

Immune checkpoint inhibitors (ICIs) enhance the immune system’s ability to target and eliminate cancer cells effectively, thereby improving the chances of long-term survival in patients with metastatic disease [72,75]. The response to ICIs is influenced by a range of factors, including tumour type and host-related factors. Recent studies have highlighted differences in tumour response rates among patients with obesity.

Several multicentre studies have found that obesity is associated with improved clinical outcomes, including progression-free survival and overall survival, in patients treated with ICIs [76,77]. One study of metastatic melanoma suggests a consistent and significant survival advantage for patients with obesity, compared to moderately improved outcomes in patients who were overweight [77]. These findings may be attributed to several factors, including higher expression of programmed cell death protein 1 (PD-1) and its ligand (PD-L1) in patients with obesity, which may enhance ICI binding and response [72]. Additionally, the microbiota profile in patients with obesity has been hypothesised to positively influence the response to ICIs [72]. Another study that observed better responses to ICIs in patients with obesity further stratified the results by sex, suggesting that men may benefit more from ICI therapy than women [78]. Further research is needed to fully explain this sex-related difference in response.

Conversely, a study of renal cancer involving patients with obesity found a median reduction in progression-free survival by 6.5 months and overall survival by 12 months, compared to patients with normal weight [89]. This suggests that the impact of obesity on ICI treatment may vary across different cancer types, indicating a complex relationship.

ICIs are not without their toxicities or immune-related adverse events (irAEs). These irAEs can be acute or chronic, with chronic irAEs potentially arising from the prolonged therapeutic effects of ICIs that extend beyond their half-life [75]. While irAEs can impact almost any organ system and vary in severity, chronic irAEs are primarily associated with the endocrine and rheumatological systems [75]. Patients with overweight or obesity tend to have a higher incidence of irAEs [76]. A meta-analysis involving cohorts from Europe, the USA, and Asia found a significant association between obesity and the development of irAEs [90]. Additionally, a retrospective study suggested that sarcopenic obesity (the combination of obesity with reduced muscle mass and function) is linked to early acute toxicity in patients treated with nivolumab or pembrolizumab [78]. Another study found that elevated BMI, in the presence of fewer than two metabolic comorbidities (such as diabetes, hypertension, or hyperlipidaemia), was significantly associated with irAEs, whereas normal BMI in patients with two or more metabolic comorbidities showed no such association [91]. This further underscores the significance of obesity in the development of irAEs. The exact mechanism through which BMI affects the development of irAEs is not fully understood. This effect is likely linked to the relationship between obesity and low-grade systemic inflammation, which may increase the risk of developing irAEs [90]. However, a recent study found no difference in the occurrence of irAEs between patients with or without obesity [91]. With only 115 (28%) out of 409 patients classified as obese, the study’s findings may have been impacted upon by an insufficient sample size [91].

## 5. Weight Loss Interventions and Cancer Risk

The overwhelming body of evidence linking obesity to cancer incidence raises the question of whether weight loss reduces cancer risk. While it may seem intuitive that weight loss would lower the risk of all types of cancer, research has yielded mixed results.

### 5.1. Bariatric Surgery and Cancer Risk

There is ample evidence that weight reduction interventions, including dietary changes, structured exercise, pharmacotherapy, and bariatric surgery, are associated with a reduced risk of cancer, particularly in individuals with severe obesity [5,92]. Among these interventions, bariatric surgery offers the greatest benefit in lowering cancer risk due to its effectiveness in achieving rapid weight loss and maintaining long-term weight reduction [5,92]. The SPLENDID (Surgical Procedures and Long-term Effectiveness in Neoplastic Disease Incidence and Death) study demonstrated a significant association between bariatric surgery and a reduced risk of obesity-related cancers (hazard ratio, 0.68) as well as cancer-related mortality (hazard ratio, 0.52). The strongest correlation was observed in patients with endometrial cancer [93]. Although the exact mechanism by which surgery reduces cancer risk is still an evolving area of research [92], it has been demonstrated that it improves high-density lipoprotein (HDL) functionality and leads to a significant reduction in systemic and adipose tissue inflammation, thereby limiting carcinogenesis [93,94].

However, a previous comprehensive review found no association between weight loss and cancer risk [95]. The retrospective design of studies, reliance on self-reported weight data, inability to determine whether weight loss was caused by cancer or a pre-existing illness, and inadequate follow-up periods may explain the observations. The absence of a link between weight loss and cancer risk reduction may also be attributed to patients having cancers that are non-obesity related, as identified by the IARC [96].

There also appears to be a disparity in cancer risk reduction between sexes [97]. Bariatric surgery has been associated with a decrease in cancer incidence in women, but not in men [92,97]. These sex-specific variations may arise from the complex interaction between hormones, body fat distribution patterns, and sex-related metabolic processes [95,97]. For example, obesity is closely linked to elevated oestrogen levels, which in turn increase the risk of hormone-sensitive cancers such as breast and endometrial cancers [95,97]. The sharp decline in oestrogen levels following weight loss, particularly in post-menopausal women whose primary source of oestrogen is fat tissue, may help explain the more favourable risk reduction observed in women [95]. Additionally, the higher accumulation of visceral fat in women, which is associated with an increased cancer risk, could also contribute to these sex differences [95]. Moreover, the limited number of weight loss studies conducted in men may also explain the absence of a similar effect in men [95].

### 5.2. Use of GLP-1 Receptor Agonists in Obesity and Association with Cancer

Glucagon-like peptide-1 receptor agonists (GLP-1RAs) are a class of medications used in the treatment of type 2 diabetes mellitus (T2DM) and/or obesity. GLP-1RAs bind to the GLP-1 receptors within the central nervous system, pancreas, and gastrointestinal tract and act to increase satiety and reduce appetite, leading to weight loss [98].

A retrospective cohort study examined the risk of developing colorectal cancer in over 1.2 million patients with T2DM who were prescribed GLP-1RAs, insulin, metformin and other oral anti-diabetic medications between 2005 and 2019. The analysis, adjusted for confounding factors such as age, sex, and BMI, showed that GLP-1RAs were associated with a statistically significant reduced risk of colorectal cancer in patients with T2DM. There was a more pronounced effect in the reduction in risk in patients with T2DM who had a BMI > 25 kg/m^2^ [99].

A further US cohort study analysed health records of 1.6 million patients with T2DM who had been prescribed GLP-1RAs, insulin, and metformin between 2005 and 2018. The study demonstrated that patients treated with GLP-1RAs compared to insulin had a statistically significant reduction in risk of developing 10 out of 13 obesity-associated cancers, including pancreatic, oeseophageal, and ovarian cancer. Compared to metformin, there was no statistically significant reduction in the risk of obesity-associated cancers in patients with T2DM prescribed GLP-1RAs, but there was an increased risk of kidney cancer [100].

A potential increased risk of development of thyroid cancer has previously been identified in pre-clinical trials for GLP-1RAs (in particular, Liraglutide) using animal models [101]. In 2023, a nationwide case–control analysis using the French national healthcare database was undertaken to further explore the risk between GLP-1RA use and the development of thyroid cancer. A cohort of patients with T2DM treated with GLP-1RAs and other antidiabetic medications excluding insulin monotherapy between 2006 and 2018 were used. From this cohort, a total of 2562 cases of thyroid cancer were identified and were matched with control subjects to account for age, gender, and length of diagnosis of T2DM. The analysis showed that there was an increased risk of all thyroid cancer and medullary thyroid cancer (MTC) associated with the use of GLP-1RAs, in particular with longer-term treatment (1–3 years). There was a higher prevalence of MTC in the cohort population than the general population [102]. However, the study did not account for obesity, which, alongside T2DM, is a risk factor for the development of thyroid cancer. The study was subject to detection bias due to increased monitoring of thyroid function and more potential for neck imaging in patients taking GLP-1RAs due to symptoms experienced as a result of both the medication and T2DM and potential obesity [103].

Furthermore, a meta-analysis of 37 randomised controlled trials of GLP-1RAs demonstrated that there was no increased risk of developing pancreatic, thyroid (including medullary thyroid), and colorectal cancer. However, the RCTs had a relatively short duration of follow-up and further long-term studies are needed [104].

In summary, based on the existing evidence, there is no requirement to avoid using GLP-1RAs due to the risk of thyroid cancer, apart from in patients with a personal or family history of medullary thyroid cancer or multiple endocrine neoplasia—this is already well-established as a contraindication.

## 6. Obesity as a Presenting Feature of Cancer

The diagnosis of cancer is often associated with weight loss. However, there are key cancer presentations in which weight gain may be the predominant symptom. Patients with paraneoplastic Cushing’s syndrome, for example, may present with weight gain as a key manifestation [105]. Furthermore, insulinomas (including metastatic insulinomas) may include weight gain as a key symptom that is reported by patients [106]. Patients who develop ascites can sometimes misreport the increased abdominal circumference as increased adiposity [107], and so any patient in whom there has been unexplained and rapid weight gain should have a thorough assessment which includes within the differential the possibility of a new cancer syndrome.

## 7. Conclusions and Future Directions

There is an increasing body of evidence relating obesity with cancer. Obesity causes pathological changes to the hormonal and inflammatory environment within the body, which in turn increases the risk of a person developing cancer. However, further research underpinning the exact pathophysiological mechanisms that belie the strong association between obesity and cancer is still needed. There is still a relatively scarce body of evidence focused on how the presence of obesity might impact upon cancer outcomes; studies examining this issue may help in defining holistic cancer management strategies. The recognition of obesity as a principal risk factor for cancer, especially the 13 obesity-associated cancers, is important to drive public health initiatives to (i) focus on the prevention of obesity (and thus cancer) and (ii) treat obesity with effective therapeutic interventions.
